# The Use of Herbal Remedies among Mothers of Young Children Living in the Central Appalachian Region

**DOI:** 10.1155/2017/1739740

**Published:** 2017-11-02

**Authors:** Monira Alwhaibi, Rashmi Goyat, Kimberly M. Kelly

**Affiliations:** ^1^Department of Clinical Pharmacy, School of Pharmacy, King Saud University, Riyadh, Saudi Arabia; ^2^Department of Pharmaceutical Systems and Policy, School of Pharmacy, West Virginia University, Morgantown, WV, USA; ^3^Mary Babb Randolph Cancer Center, West Virginia University, Morgantown, WV, USA

## Abstract

**Introduction:**

Women often use herbal remedies as a complement or alternative to traditional medicine. Guided by the Comprehensive Model of Information Seeking, this study examined use of herbal remedies among mothers of young children living in the Central Appalachian Region.

**Methods:**

A cross-sectional study was conducted among mothers of young children (*n* = 178). The outcome measure of interest was the use of any herbal remedy in the past six months. Two scales were developed to measure information seeking channels and to measure beliefs about the safety/efficacy of herbal remedies.

**Results:**

One-third reported using herbal remedies in the past six months, with fenugreek being the most common. Most reported using herbal remedies to increase breast milk production and to relieve cold/flu-like symptoms. Women scoring highest in information seeking channels were three times as likely to use herbal remedies. Women scoring highest in the beliefs about the safety/efficacy of herbal remedies were four times as likely to use herbal remedies.

**Conclusion:**

Herbal remedies are commonly used among women living in the Central Appalachian Region, a region with lower education and income level. Therefore, public health interventions about the types, safety, and efficacy of herbal remedies may improve health within this population.

## 1. Introduction

Herbal remedies are a subset of herbal products and are the most commonly used complementary and alternative medicines (CAM) by adults in the United States (US) [1, 2]. An estimated 38.2 million adults in the USA use herbal products and dietary supplements [1, 2]. The use of herbal remedies is higher among women as compared to men (21.0% versus 16.7%) [1]. Women commonly use herbal remedies during pregnancy, lactation, and postpartum periods [3–8]. Herbal remedied are used by many women during the lactation period to increase milk production (galactagogues) [9]. The most commonly used galactagogues are fenugreek, goat rue's leaf, milk thistle, and the root of wild asparagus [9]. In spite of the use of these herbal remedies in young mothers, the way women seek information about these agents is largely understudied.

Studies found that women use herbal remedies as a preventative measure or as a treatment due to personal dissatisfaction with conventional treatment, side effects of prescribed medications, and history of treatment failure [7, 8, 10]. In addition, other contributing factors for the use of herbal remedies among women include easy access, recommendation from family and friends, availability of information about the herbal remedies, perceived efficacy and safety of herbal remedies, and women's information seeking behavior [7, 9–11]. Information seeking behavior is purposively seeking information in order to achieve a goal [12]. Information seeking behavior and beliefs were identified as important factors that affect the use of healthcare services and CAM in the wider literature [13–15].

To guide a comprehensive understanding of factors that influence the use of herbal remedies, we used the Comprehensive Model of Information Seeking (CMIS) [16]. This model has been widely used in the literature to assess adherence to medications, treatment decisions, and other health outcomes [17]. The CMIS model posits that four antecedents (i.e., factors that predispose individuals to seek information) including the demographic factors,* experience*,* salience,* and* beliefs *can affect information carrier characteristics (i.e., how people seek information).* Channels (information sources) also* influence information seeking action (i.e., use of herbal remedies). The current study measured four antecedents:* demographics* or background characteristics of the participants (e.g., age, race),* experience* or their prior contact (perceived sensitivity to medications),* salience *or personal relevance factors (mood), and* beliefs* about the safety and efficacy of herbal remedies for their role in information seeking action. We also examined information carrier characteristics in the form of the* channel* or information source. Although the factors that affect the herbal remedies use have been examined widely in the literature, we identified no studies utilizing the CMIS to understand herbal remedy use and information seeking in mothers of young children. These mothers are making critical decisions about the care of their children, which may ultimately impact their growth and development. In addition, women may perceive herbal remedies as less harmful to their children. Thus, women of young children are important for study with regard to herbal remedies.

There is also a knowledge gap on the use of herbal remedies among women living in the Central Appalachia. Appalachia is a region that experiences lower education and income level, lack of health coverage, and a shortage of health care providers as compared to the other regions in the United States. It has been reported that the Central Appalachian Region, including WV, has the worst economic status of all the Appalachian regions [18]. CAM use in the Appalachian region has been reported higher than the general national population [19]. The prevalence of CAM use in the Appalachian region ranges from 45 to 56% as reported by some studies [20, 21]. CAM use within the Appalachian population includes folk medicine and spiritual practices like prayer [19]. In addition, there is abundant research on health disparities, inadequate access to health care, and distrust of formalized medical systems among this community [20]. However, there is a paucity of research on the use of herbal remedies among women with young children in this community. In addition, the impact of beliefs and information seeking* channels* on the use of herbal remedies among women living in the Central Appalachian Region has not been examined before. Therefore, the objectives of this study were to (1) examine herbal remedy use among women living in the Central Appalachian Region and (2) describe the factors that may play a role in the use of herbal remedies.

## 2. Methods

### 2.1. Participants

Women participating in this study met the following inclusion criteria: aged 18 years and above, living in the Central Appalachian Region, have at least one child under three years of age, and able to read and write in English. Of women who completed the survey (*n* = 226), 78.8% (*n* = 178) met inclusion criteria.

### 2.2. Procedures

The survey was constructed after extensive review of the literature to identify existing survey and instruments. An initial draft of the survey and instruments was developed. The initial draft was comprised of demographics, a new item that measures herbal remedies use in the past six months, an existing sensitivity to medications scale, and newly developed scales to measure information seeking channels and the beliefs about the efficacy and safety. Then, a group of investigators (*n* = 8) reviewed the survey and the instruments for content, clarity, and face validity. The survey, advertisements, and consent form were approved by the Institutional Review Board (IRB) of West Virginia University. After IRB approval, the survey was pilot tested, and content validity was assessed by conducting cognitive interviews among a purposive sample of women with diverse ages and races (*N* = 8). These women received a university-themed gift as an incentive for participation. Modifications to the survey were completed in response to the cognitive interview. After the survey had been discussed among the investigators, revisions were submitted to and approved by the IRB. The survey was then uploaded on web-based survey software, Survey Monkey, and women were recruited from several sites including social media sites (e.g., Facebook breastfeeding groups), listserv emails, daycares, and physician's offices. Each eligible woman was asked to complete an informed consent form before proceeding to complete the survey.

### 2.3. Measures

#### 2.3.1. Dependent Variable

*Information Seeking Action* . Use of herbal remedies in the past six months (i.e., information seeking action) was measured with a single item. Participants were asked the following question: “have you used any herbal remedy in the past six months? (e.g., Goat's rue leaf, German chamomile, ginger, fenugreek, etc.).” If they responded yes, then they were asked about the type of herbal remedies and the health condition for which it was used.

#### 2.3.2. Independent Variables

*Antecedents* . Antecedents included demographic factors,* experience*,* salience,* and* beliefs*.* Demographic* factors included age, race (White, others), ethnicity (Hispanic, others), marital status (married, others), education (equal to/less than Bachelor's degree, and greater than Bachelor's degree), income (less than $50,000, equal to/greater than $50,000), employment (full time, part time/unemployed), insurance (insured/uninsured), Appalachian identity (yes, no), and religious affiliation (Christian, non-Christian).

*Experience* was measured by Perceived Sensitivity to Medications (PSM), which was measured using a five-item validated Likert scale [22].* Salience* included mood using the short form of the Profile of Mood Scale (POMS) [23]. In this scale, depression and anxiety items were averaged to create a mean score of 1 to 5, where 1 is not at all and 5 is very much [23].* The scale of beliefs* about herbal remedies was developed after extensive review of the literature to identify existing scales (Table 1). The scale of beliefs about herbal remedies was designed to measure two major constructs: beliefs about the efficacy and beliefs about the safety of herbal remedies. Participants were asked to rate whether they believed that herbal remedies were more safe and effective than prescription medicines on a 5-point Likert scale, where 1 was “strongly disagree” and 5 was “strongly agree.” A mean score was computed, which could range from 1 to 5, with a higher score indicating higher beliefs about the efficacy and safety of herbal remedies.

*Information Carrier Characteristics* . Information carrier characteristics were the specific* channels* used to seek information. A six-item 5-point Likert scale was developed to measure how often women were likely to use a particular source of information to seek information about herbal remedies. The information seeking channels scale (Table 1) was designed to measure the extent of seeking information about the herbal remedies. Thus, it is a composite measure of the types of information sources used, as well as their behavioral frequency of use. For ease of explanation, we will refer to this as* channel*. This scale had six items; items were scored on a 5-point Likert scale ranging from 1 = never to 5 = always. A mean score was computed for each participant, which could range from 1 to 5, with higher scores indicating higher information seeking* channel* about the herbal remedies.

### 2.4. Analysis Plan

*Beliefs* about the efficacy and safety of herbal remedies and information seeking* channels* scales were tested for construct reliability and validity. Reliability was assessed using Cronbach's alpha for measuring internal consistency. Principle Component Analysis (PCA) with varimax rotation was used to examine the construct validity and explore factor structure. Criteria for the number of factors extracted include eigenvalues greater than one. After identifying the number of factors, a factor loading greater than 0.4 was used as a cutoff point for defining items associated with a given factor, and we have also allowed for greater than 0.2 differences so that our items cannot load heavily on two factors.

Descriptive statistics were used to describe our sample. The dependent variable was the use of any herbal remedy in the past six months, which was analyzed as a binary variable (yes/no). Independent variables included* demographic* factors (age, race, ethnicity, marital status, education, income, employment, insurance, the Appalachian region, and religion), experience (mean sensitivity to medication),* salience* (mean mood),* beliefs* (mean beliefs about the efficacy and safety of herbal remedies), and* channels* (mean information seeking).* t*-test and chi-square statistics were used to test the unadjusted association between herbal remedy use and the independent variables. Factors that were significant at *p* < 0.05 were retained in the binary logistic regression model, which was used to test the adjusted association between the herbal remedy use and the independent variables. All analyses were conducted using the Statistical Analysis System Software (SAS® 9.4 Institute Inc., Cary, NC, USA).

## 3. Results

### 3.1. Reliability and Construct Validity

The Cronbach alpha for measuring the internal consistency for the* beliefs* scale (Cronbach's alpha = 0.87) and the information seeking* channels* scale (Cronbach's alpha = 0.63) indicates that our instruments were reliable (Table 1).

### 3.2. Description of the Study Sample

Table 2 describes the total study sample characteristics; the mean age of women participating in our survey was 31.3 (SD = 4.7). The majority of the sample was White (94.4%), non-Hispanic (98.3%), and married (88.2%); had a Bachelor's degree or less (53.9%) and greater than $50,000 income (82.3%); and were insured (92.1%) and full-time employed (61.4%). A high proportion of the sample identified themselves as being from the Appalachian region (40.7%). In terms of* channels*, the Internet was the most frequently used (M = 3.2), followed by friends/coworkers (M = 2.7), family (M = 2.5), printed materials (M = 2.5), health care providers (M = 2.2), and television (M = 2.1).

### 3.3. Factors Affecting CAM Use

We found that 33.5% of women reported using herbal remedies in the past six months.  Table 3 describes the commonly reported herbal remedies. We found that fenugreek (35.1%) was the most widely used herbal remedy in our study sample followed by honey (8.8%), chamomile (5.3%), and ginger (5.3%).  Table 3 also describes the reported reasons for herbal remedies use; herbal remedies were mainly used to stimulate or increase lactation (38.6%) and to treat cold/flu symptoms (26.3%).

Herbal remedies use among the study sample was significantly associated with information seeking* channels*, employment, and* beliefs* about the efficacy and safety of herbal remedies (Table 4). Women who scored highest in information seeking* channels* were three times as likely to use herbal remedies as compared to those with the lowest information seeking* channels* (OR = 3.61, 95% CI = 1.433–9.77). Women who scored highest in the* belief* in the safety and efficacy of the herbal remedies were four times as likely to use herbal remedies as compared to those with the lowest belief in herbal remedies safety and efficacy (OR = 4.02, 95% CI = 1.468–9.57). We also found that women who we were part time/unemployed were 2.98 times as likely to use herbal remedies as compared to those who were full-time employed (OR = 2.98, 95% CI = 1.05–8.47).

## 4. Discussion

This study examined the use of herbal remedies among women living in the Central Appalachian Region, particularly in women with young children. We found that one-third of women in our study sample reported the use of herbal remedies in the past six months. The prevalence of herbal remedies use from our study is lower than the rate reported from a study conducted among women in the Central Appalachian Region (33.5% versus 56.0%) [19]. It is important to note that women in the previous study were from one county in WV who were seeking free care and may not be representative of the broader population. Our sample had higher income and was not from a clinical population.

Guided by the CMIS model, this study has also examined the differences in the use of herbal remedies in terms of demographic factors, beliefs, and information seeking* channels*. As suggested by the CMIS model, we found that having greater belief in the efficacy and safety of herbal remedies was associated with the use of herbal remedies. This is not surprising since women often consider the herbal remedies as “natural,” and therefore they consider them to be safe. The most commonly reported herbal remedies by women in our study were fenugreek followed by honey and chamomile. Fenugreek has established safety and efficacy and was commonly used to increase milk production [24]; therefore women may have used it to increase milk production. In fact, the majority of women reported using the herbal remedies to increase breast milk production and for cold/flu-like symptoms.

Within the CMIS model, information seeking channels are an important factor that women may use to guide them to select the herbal remedies for self-management. Thus, this study has found that information seeking channels were associated with the use of herbal remedies. In the wider literature, individuals' information seeking behavior is associated with higher use of healthcare services and engagement in healthy behaviors [13–15]. Women may seek information to complement information received from physicians [25]; however, in our study, it appeared that the Internet and family and friends were more common channels. Healthcare providers can play an important role in providing information about the use of herbal remedies and also in providing feedback about channels (including the Internet) of good quality information to learn more about herbal remedies. Further, important sociodemographic factors within the CMIS model such as education, income, and Appalachian identity were not associated with herbal remedies use in our sample except for employment status; unemployed people had a higher herbal remedies use as compared to employed people. This is consistent with prior studies reporting that herbal remedies use among individuals in the Appalachian community is related to poverty and poor access to healthcare [18].

Although our study identified important factors that affect the use of herbal remedies among women living in Central Appalachian Region, our findings suggest the need for further research to examine what type of health information women seek, and qualitative methods may help to learn more about specific information practices of women of young children. This study has many advantages. This is the first study that examined the herbal remedies use among women living in the Central Appalachian Region. Our study added to the nascent literature new validated scales to measure the beliefs about the efficacy and safety of herbal remedies and information seeking channels about herbal remedies. However, our study has some limitations. Our sample consisted mostly of women in the Appalachian West Virginia; thus, the study findings might differ across Appalachia. Also, we cannot exclude self-selection bias for women who participated in the study, as the study was Internet-based. All measures in the study were self-reported and hence subject to recall bias, which may be more or less relevant due to the variable considered. Further, we did not assess if the use of herbal remedies was regular or sporadic. Despite these limitations, this is the first study of its kind that used validated information seeking* channels* scale and beliefs scale to assess the use of herbal remedies among women living in the Central Appalachian Region.

## 5. Conclusion

In conclusion, about one-third of women reported the use of herbal remedies in the past six months. Consistent with the CMIS model, higher beliefs about the efficacy and safety and higher information seeking channels were associated with herbal remedies use. Public health interventions and healthcare providers' communication are needed to increase the awareness about the safety and efficacy of many herbal remedies.

## Figures and Tables

**Table 1:** Factor loadings of the items for the belief's in efficacy and safety of herbal remedies scale and information seeking channels scale.

Items	Factor loading	Cronbach alpha
*Beliefs about the safety and efficacy of herbal remedies*		
Please indicate how much you agree or disagree with the following statements:		0.87
(1) Herbal remedies are not harmful.	0.67	
(2) Herbal remedies are safe for use in children.	0.85	
(3) Herbal remedies are effective.	0.79	
(4) Herbal remedies have fewer side effects than prescription medication.	0.88	
(5) Herbal remedies help body's immune system.	0.88	

*Information seeking channels about the remedies*		
Please rate how often have you heard about herbal remedies from the following sources:		0.63
(1) Printed material (Newspaper, book, or magazine).	0.70	
(2) Television.	0.44	
(3) Internet.	0.79	
(4) Family.	0.74	
(5) Friends or coworkers.	0.70	
(6) Health care provider (physician, pharmacist, nurse).	0.24	

*Note*. Principle Component Analysis (PCA) for the belief scale revealed that all of our five items were highly loading on one factor; this factor accounts for 68.30% of the variance. PCA for information seeking channels scale revealed that five items (printed material, television, Internet, family, friends, and coworkers) were highly loading in one factor (>0.4); this factor accounts for 47.01% of the variance.

**Table 2:** Description of the study sample. Number and raw percentage by herbal remedies use.

	Total sample		Herbal remedies use		No herbal remedies use		
	*N*	%	*N*	%	*N*	%	Sig
*Total*	178	100	57	33.5	121	66.5	
Age (mean (SD))	31.3 (4.7)		30.4 (4.8)		32.4 (5.2)		
Race							
White	168	94.4	52	32.7	108	67.3	
Others	10	5.3	5	50.0	5	50.0	
Ethnicity							
Hispanic	3	1.7	1	37.0	3	63.0	
Others	175	98.3	30	30.0	145	70.0	
Marital status							
Married	157	88.2	50	33.8	99	66.2	
Others	21	11.8	7	33.3	14	66.7	
Education							
Equal/LE Bachelor's degree	95	53.9	32	35.2	59	64.8	
GE Bachelor's degree	81	46.1	25	32.1	54	68.0	
Income							
LE 50,000	31	17.7	9	30.0	21	70.0	
Equal to/GT 50,000	146	82.3	47	34.1	92	65.9	
Employment							*∗∗∗*
Full time	110	61.4	21	20.4	89	79.6	
Part time/unemployed	68	38.6	36	54.6	32	45.5	
Insurance							
Insured	164	92.1	50	32.1	107	68.0	
Uninsured	14	8.0	7	53.9	6	46.2	
Appalachian							
Yes	72	41.1	26	37.7	43	62.3	
No	105	58.9	30	30.3	70	69.7	
Religion							
Christian	132	73.9	43	34.4	83	65.6	
Non-Christian	46	26.1	14	31.8	30	68.2	
Breastfed							
Yes	155	92.8	50	34.4	105	65.6	
No	12	7.2	4	8.3	8	91.7	
Sensitivity (mean (SD))	1.63 (0.6)		2.64 (0.93)		2.43 (0.77)		
Mood (mean (SD))	1.90 (0.8)		1.98 (0.94)		1.78 (0.92)		
Belief (mean (SD))	2.03 (0.5)		3.37 (0.68)		2.82 (0.63)		*∗∗∗*
Information seeking channels (M (SD))	1.72 (0.4)		2.84 (0.48)		2.38 (0.61)		*∗∗∗*

*Note.* Based on 178 women, aged over 18 years and having at least one child less than two years old. Asterisks represent significant baseline characteristic by herbal remedies use based on chi-square tests. GT: greater than; LT: less than; SD: Standard Deviation. ^*∗∗∗*^*p* < .001; .001 < ^*∗∗*^*p* < .01; .01 < ^*∗*^*p* < .05.

**Table 3:** Number and percentage of the reported herbal remedies and reasons for herbal remedies use among herbal remedies users.

	*N*	%
Reported herbal remedies		
Fenugreek	20	35.1
Honey	5	8.8
Chamomile	3	5.3
Ginger	3	5.3
Other herbal remedies	33	45.5
Reported reasons for herbal remedies use		
Increase lactation	22	38.6
Treat cold/flu symptoms	15	26.3
Others	25	35.1

*Note*. Based on 178 women, age over 18 years, with at least one child less than two years old. Other herbal remedies included *Echinacea*, doterra, oregano, peppermint, herbal tea, mothers milk tea, elderberry syrup, probiotics, and *Vitex*.

**Table 4:** Odds ratios and 95% confidence intervals from binary logistic regression on any herbal remedy in the past six months.

Variable	Any herbal remedy use		
	OR	95% CI	Sig
Information seeking channels			
	3.61	1.33–9.77	*∗*
Belief			
	4.02	1.68–9.57	*∗* *∗*
Employment			
Full time (Ref.)			
Part time/unemployed	2.98	1.05–8.47	*∗*

*Note*. Based on 178 women, age over 18 years, with at least one child less than two years old. Asterisks represent significant group differences compared to reference group (no herbal remedies use in the past 6 months) based on binary logistic regression; OR: Odds Ratio, CI: Confidence Interval, and Sig: significance.
